# The Role of Patient-Reported Social Factors in Promoting Buprenorphine Consistency

**DOI:** 10.33696/psychiatry.1.006

**Published:** 2023

**Authors:** Brenna Cook, Michelle Eglovitch, Dace Svikis, Caitlin E Martin

**Affiliations:** 1 School of Medicine, Virginia Commonwealth University, Richmond, VA 23298, USA; 2 Department of Psychology, Virginia Commonwealth University, 23298, USA; 3 Department of Obstetrics and Gynecology, Virginia Commonwealth University, Richmond, VA 23298, USA; 4 Institute for Drug and Alcohol Studies, Virginia Commonwealth University, Richmond, VA 23298, USA

**Keywords:** Opioid use disorder, Buprenorphine, Substance use disorder, Medication for opioid use disorder, Social factors, Social determinates of Health, Treatment

## Abstract

**Background:**

While medications for opioid use disorder (MOUD) reduce overdose risk, inconsistent use can lead to substance use recurrence and compromise achieving optimal opioid use disorder (OUD) treatment outcomes. Research is limited on patient-reported perspectives on consistency of MOUD self-administration at home and its related social factors.

**Objectives:**

The primary aim was to report on rates of patient-reported buprenorphine consistency among a sample receiving outpatient OUD treatment. The secondary aim was to explore differences in social determinants of health (SDOH) between patients reporting and not reporting lapses in buprenorphine dosing.

**Methods:**

This is a secondary analysis from a cross-sectional survey and medical record abstraction study (N=96). The primary outcome was patient-reported buprenorphine consistency, as defined as no lapses in buprenorphine dosing in a preceding 28-day period. SDOH survey items were adapted from the Healthy People 2030 framework.

**Results:**

Participants (n= 96) were three quarters female (74.0%); most identified as white (54.2%) or Black (38.9%). Most reported not missing any buprenorphine doses over the preceding 28-days (88.5%). Demographic and clinical variables were similar between buprenorphine consistency groups. Participants reporting no missed doses reported few negative social determinants of health (examples: 90% not needing help reading hospital materials and not being afraid that they would be hurt in their apartment building or house).

**Discussion:**

These findings reinforce the known role of SDOH as strong predictors of treatment outcomes for chronic diseases (like substance use disorders), beyond contributions by demographic or clinical variables alone.

**Conclusions:**

Future MOUD research should incorporate patient perspectives with the goal of informing patient-centered interventions.

**Scientific Significance:**

Promoting consistency in buprenorphine dosing using strategies grounded in patient experience could be an avenue to promote positive OUD treatment outcomes.

## Introduction

Opioid Use Disorder (OUD) is a chronic, relapsing disease that causes clinically significant impairment, negatively impacting diverse patient populations [1]. Opioid-related deaths increased by 345% between 2001 and 2016 [2]. Medications for opioid use disorder (MOUD), such as buprenorphine and methadone, are effective treatments to decrease overdose risk and promote positive health outcomes [3]. However, to gain optimal benefits from MOUD treatment, daily MOUD dosing is required, as inconsistency in MOUD use can lead to withdrawal symptoms, cravings, substance use recurrence [4], and, ultimately, treatment discontinuation and heightened overdose risk [5].

Buprenorphine is unique, as patients typically self-administer buprenorphine at home after filling a prescription from their outpatient provider [6]. Thus, lapses in buprenorphine dosing may occur due to many challenges inherent to the patient and their environment. Prior studies have found that factors such as age and mental health comorbidities are associated with decreased adherence to MOUD [7,8]. While previous research has examined patient perspectives in relation to OUD treatment broadly, research prioritizing patient perspectives on social factors in the context of daily buprenorphine administration is limited. Additionally, the role of social factors in promoting buprenorphine consistency in the patient’s home setting is less well understood. Such data could guide improvements in the quality of OUD treatment in the ongoing overdose crisis.

The primary aim of this study is to describe rates of patient-reported buprenorphine consistency among a sample of patients receiving outpatient OUD treatment, defined as no lapses in buprenorphine dosing in a preceding 28-day period. The secondary aim is to explore differences in demographics, clinical variables, and social determinants of health between patients reporting and not reporting lapses in buprenorphine dosing.

## Methods

### Participants and study design

This is a secondary analysis of data collected from an ongoing cross-sectional survey and medical record abstraction study investigating the relationship between sleep and recovery from OUD. The parent study, conducted by our investigative group from February 2022 to September 2023, enrolled non-pregnant patients between the ages of 18–65 stabilized on buprenorphine from an outpatient addiction medicine treatment clinic affiliated with an academic institution in a southern, Medicaid expanded state. More detailed methods for the parent study are described elsewhere [9].

The clinic utilizes a comprehensive, recovery-oriented care model providing MOUD as well as integrated on-site health services. Research assistants directly approached eligible patients in the clinic, confirmed eligibility, and invited eligible patients to participate in the study.

### Demographic, clinical, and social determinants of health variables

Demographic items obtained from the survey and/or chart abstraction included age, gender, race, insurance status, education, and employment. Clinical variables obtained from the survey and/or chart abstraction included duration of OUD treatment, daily buprenorphine dose, and mental health diagnoses. Survey items reflecting social determinants of health were adapted from the five domains reflected in the Healthy People 2030 framework [10]: education, economic stability, neighborhood/built environment, health and healthcare, and social and community support. Lastly, participants completed the Substance Use Recovery Evaluator (SURE), a validated questionnaire assessing recovery capital, defined as the total number of resources an individual has that promotes initiation and maintenance of recovery [11,12]. Participants completed the 26-item questionnaire, which uses a three- and five-point scoring system. Scores range from 21–63, where higher scores are associated with increased recovery capital.

### Buprenorphine consistency

Buprenorphine consistency, as defined by not missing any doses of buprenorphine over a preceding 28-day period, was determined using an adapted timeline follow-back interview [13].

### Data analysis

In line with the research question for our secondary aim, we explored differences in descriptive variables between groups defined by our primary outcome of patient reported buprenorphine consistency. Specifically, univariate comparisons were made using Pearson chi-square for categorical variables and T test for continuous variables. Data analysis was conducted using SPSS version 28 [14].

## Results

Participants (n= 96) were approximately three quarters female (74.0%) and aged 40.85 ± 10.35 years (Table 1). All participants identified as cisgender. Most patients identified as White (54.2%) or Black (38.9%). The majority of participants were unemployed (64.5%) and utilized public health insurance (94.1%).

The primary outcome was patient-reported buprenorphine consistency. Most participants reported that they did not miss any doses of buprenorphine over the preceding 28-day period (88.5%) (Table 1). Notably, participants who missed doses of buprenorphine reported losing or running out of buprenorphine as the most common reasons for the lapse (Table 2).

Demographics were generally similar between buprenorphine consistency groups. Regarding clinical variables, participants reporting no missed doses of buprenorphine demonstrated a longer mean duration of buprenorphine treatment than those who reported missed dose(s) (23.7 months vs 5.6 months) (Table 1).

Participants reporting no missed doses of buprenorphine generally also reported few negative social determinants of health. For example, approximately 90% reported not needing help reading hospital materials, and not being afraid that they would be hurt in their apartment building or house. Regarding stable housing, 63.3% of patients self-reporting missed doses of buprenorphine answered that they were worried that in the next 2 months they may not have stable housing. Additionally, recovery capital scores were generally similar between groups.

## Discussion

In this sample of patients in outpatient OUD treatment, over 80% self-reported consistency in their buprenorphine dosing. In our exploration of differences between these patients and their counterparts reporting buprenorphine lapses, demographic and clinical factors generally were similar. However, challenging social determinants of health were not as prevalent in buprenorphine consistency patients. Overall, these findings highlight potential areas of intervention for OUD treatment programs to promote consistency in buprenorphine dosing using strengths-based approaches.

Our study found high rates of self-reported buprenorphine consistency among participants. While prior literature examining day-to-day buprenorphine consistency is limited, previous studies have investigated buprenorphine consistency through different measures, including insurance claims data and prescription drug monitoring programs. One prior study that investigated buprenorphine adherence through prescription drug monitoring found that 26% of patients were adherent to buprenorphine after 180 days, with adherence defined as greater than or equal to 80% of days covered over a 180-day period as determined by examining pharmacy claims and dispensing dates [15]. Similarly, another study reported a 40.8% adherence rate over a 60-day period, with adherence defined by no gaps in prescription fills longer than 10 days [16]. While buprenorphine consistency was higher in our sample compared to previous literature, this could be due to our less rigorous definition of buprenorphine consistency, our shorter timeframe for evaluating consistency, and our use of patient-reported consistency, which may offer a unique glimpse into medication patterns and behaviors that cannot be captured by prescription monitoring alone. This is supported by a recent study that used a 7-day timeline follow back in conjunction with urine drug levels to determine short-term buprenorphine consistency over a 12-week period and found that 70% of patients were fully adherent to buprenorphine, as defined by taking buprenorphine as prescribed [4]. Future treatment outcomes research should incorporate patient-reported measures to ensure a comprehensive understanding of medication patterns.

In our exploration of differences in the prevalence of SDOH using a predefined framework, participants without buprenorphine lapses less often reported problems with violence, unstable housing, and health literacy, indicating their role as potential targets for future investigations of OUD treatment interventions. Prior studies have examined similar associations in populations treated for HIV, such as associations between decreased HIV medication adherence and challenges related to SDOH, including unstable housing, decreased health literacy, food insecurity, and intimate partner violence [17]. These findings reinforce the known role of SDOH as strong predictors of treatment outcomes for chronic diseases (like substance use disorders), beyond contributions by demographic or clinical variables alone. This predictive role of SDOH is further reinforced when examining our population’s recovery capital, which describes the total number of resources an individual has that promotes initiation and maintenance of recovery [12]. While recovery capital was high in both groups, the subset of patients reporting missed doses of buprenorphine still experienced significant challenges in relation to SDOH. Future clinical practice and medication for OUD treatment investigations should include standardized measurements of SDOH in addition to the clinical and psychosocial factors traditionally associated with recovery, to ensure holistic participant profiles are captured in which to interpret study results.

There are several limitations of our study. The small sample size from a single clinic limited the power to detect significant differences and generalizability of our findings. Social desirability bias was possible, especially given the purpose of our study which prioritized a patient-reported buprenorphine consistency outcome. Additionally, given that our definition of our outcome differs from previous research, synthesizing and incorporating our results into broader work could be a challenge and may limit the generalizability of our findings.

## Conclusion

Promoting consistency in buprenorphine dosing could be an avenue to support optimal OUD treatment and recovery outcomes. Achieving a better understanding, from the patient perspective, of potential targets to achieve this goal is warranted. Incorporating social determinants of health in OUD treatment clinical care and research could inform future individualized, patient-centered interventions to promote buprenorphine consistency.

## Figures and Tables

**Table 1.** Demographics, Clinical Variables, and Social Determinants of Health as Reported by Study Sample Participants, by Patient-Reported Buprenorphine Dosing Consistency Group.

	Self reported no missed buprenorphine doses (n=85)	Self reported missed buprenorphine doses (n=11)
Age (years, mean ± STD)	39.7 (10.6)	42.0 (10.1)
Gender		
Male	22 (25.9%)	3 (27.3%)
Female	62 (72.9%)	8 (72.7%)
Race		
Black	32 (37.6%)	5 (45.5%)
White	47 (55.3%)	5 (45.5%)
Other	6 (7.1%)	1 (9.0%)
Ethnicity		
Not Hispanic or Latino	67 (78.8%)	8 (72.7%)
Hispanic or Latino	4 (4.7%)	0 (0%)
Insurance status		
Public	72 (84.7%)	8 (72.8%)
Private	5 (5.9%)	0 (0%)
Education		
Less than high school education	32 (37.6%)	3 (27.3%)
High school education	27 (31.8%)	5 (45.5%)
More than high school education	26 (30.1%)	3 (27.3%)
Employment		
Employed	26 (30.6%)	2 (18.2%)
Unemployed	43 (50.6%)	8 (72.7%)
Co-morbid mental health conditions (% yes)	70 (82.4%)	10 (90.9%)
Duration of Treatment (months, mean ± STD)	23.7 months	5.6 months
Daily buprenorphine dose (mg, median and range)	24 (4–30)	24 (8–24)
**Education Domain**		
*Do you ever need help reading hospital materials? (% yes)*	9 (10.6%)	3 (27.3%)
**Economic Stability Domain**		
*Are you worried that in the next2 months, you may not have stable housing? (% yes)*	27 (31.8%)	7 (63.6%)
**Neighborhood/Build Environment Domain**		
*Are you afraid you might be hurt in your apartment building or house? (% yes)*	3 (3.5%)	2 (18.2%)
**Health and Healthcare Domain**		
*In the last 12 months, have you ever had to go without healthcare because you didn’t have a way to get there? (% yes)*	16 (18.8%)	4 (36.4%)
**Social and Community Domain**		
*Within the last year, have you been afraid of your partner or ex-partner? (% yes)*	6 (7.1%)	4 (36.4%)
**SURE Score (possible range: 21–63)**	51.2 (7.2)	47.8 (10.7)

**Table 2:** Patient-reported reasons for missed buprenorphine doses (n=11)

Reason	
Ran out of buprenorphine	3 (27.3%)
Lost buprenorphine	3 (27.3%)
Got distracted/forgot	2 (18.2%)
Illness	2 (18.2%)
Stolen buprenorphine	1 (9.0%)
Returned to use	1 (9.0%)
